# Virucidal activity of *Haemaphysalis longicornis* longicin P4 peptide against tick-borne encephalitis virus surrogate Langat virus

**DOI:** 10.1186/s13071-016-1344-5

**Published:** 2016-02-02

**Authors:** Melbourne Rio Talactac, Kentaro Yoshii, Hiroki Maeda, Kodai Kusakisako, Emmanuel Pacia Hernandez, Naotoshi Tsuji, Kozo Fujisaki, Remil Linggatong Galay, Tetsuya Tanaka, Masami Mochizuki

**Affiliations:** Department of Pathological and Preventive Veterinary Science, The United Graduate School of Veterinary Science, Yamaguchi University, Yoshida, Yamaguchi 753-8515 Japan; Laboratory of Infectious Diseases, Joint Faculty of Veterinary Medicine, Kagoshima University, 1-21-24 Korimoto, Kagoshima, 890-0065 Japan; Laboratory of Public Health, Graduate School of Veterinary Medicine, Hokkaido University, Kita-ku kita-18 nishi-9, Sapporo, Hokkaido 060-0818 Japan; Department of Parasitology, Kitasato University School of Medicine, 1-15-1 Kitasato, Minami, Sagamihara, Kanagawa 252-0374 Japan; Zen-noh Institute of Animal Health, Ohja, Sakura, Chiba 285-0043 Japan; Department of Clinical and Population Health, College of Veterinary Medicine and Biomedical Sciences, Cavite State University, Cavite, 4122 Philippines; Department of Veterinary Paraclinical Sciences, College of Veterinary Medicine, University of the Philippines Los Baños, Los Baños, Laguna 4031 Philippines

**Keywords:** Antimicrobial peptide, Longicin P4, Langat virus, *Haemaphysalis longicornis*

## Abstract

**Background:**

Longicin is a defensin-like peptide, identified from the midgut epithelium of hard tick *Haemaphysalis longicornis*. Several studies have already shown the antimicrobial and parasiticidal activities of longicin peptide and one of its synthetic partial analogs, longicin P4. In this study, longicin peptides were tested for potential antiviral activity against Langat virus (LGTV), a tick-borne flavivirus.

**Methods:**

Longicin P1 and P4 peptides were chemically synthesized. Antiviral activity of the longicin peptides against LGTV was evaluated through in vitro virucidal assays, wherein the antiviral efficacy was determined by reduction in number of viral foci and virus yield. Additionally, longicin P4 was also tested for its activity against human adenovirus, a non-enveloped virus. Lastly, to assess the importance of longicin on the innate antiviral immunity of *H. longicornis* ticks, gene silencing through RNAi was performed.

**Results:**

Longicin P4 produced significant viral foci reduction and lower virus yield against LGTV, while longicin P1 failed to demonstrate the same results. Conversely, both longicin partial analogs (P1 and P4) did not show significant antiviral activity when tested on adenovirus. In addition, *longicin*-silenced ticks showed significantly higher virus titer after 7 days post-infection but a significantly lower titer was detected after an additional 14 days of observation as compared to the *Luc* dsRNA-injected ticks. Mortality in both groups did not show any significant difference.

**Conclusion:**

Our results suggest that longicin P4 has in vitro antiviral activity against LGTV but not against a non-enveloped virus such as adenovirus. Likewise, though most cationic antimicrobial peptides like longicin act directly on target membranes, the exact mechanism of membrane targeting of longicin P4 in enveloped viruses, such as LGTV, requires further investigation. Lastly, while the in vitro virucidal capacity of longicin P4 was confirmed in this study, the role of the endogenous tick longicin in the antiviral defense of *H. longicornis* against LGTV still remains to be demonstrated.

**Electronic supplementary material:**

The online version of this article (doi:10.1186/s13071-016-1344-5) contains supplementary material, which is available to authorized users.

## Background

Ticks are hematophagous arachnids capable of transmitting several disease-causing pathogens in domestic and wild animals, including humans [1, 2]. As carriers of several pathogenic microorganisms, protozoa, rickettsiae, spirochaetes, and viruses [3, 4], ticks need to employ broad spectrum innate immunity mechanisms that will allow them to maintain the pathogens and commensal microbes without impairing their viability and further development [2, 5]. As previously demonstrated, antimicrobial proteins and peptides play a major role in protecting ticks against microorganisms [6, 7].

Antimicrobial peptides are ancient immune molecules that are important in invertebrate and vertebrate host defenses [8, 9]. These peptides display broad-spectrum biological activity against bacteria, yeast, fungi, protozoan parasites and enveloped viruses [10–12] and have been demonstrated to possess immunomodulatory properties [13]. Numerous small molecules such as defensins, lysozymes or by tick-specific antimicrobial compounds such as microplusin provide the direct antimicrobial defense in ticks [2].

Longicin, a defensin-like peptide identified from the midgut epithelium in the hard tick *Haemaphysalis longicornis*, is a promising cationic antimicrobial peptide. Many studies have shown that longicin and one of its synthetic partial analogs (longicin P4) have antimicrobial, fungicidal, and parasiticidal properties [5, 14, 15]. Thus, making them attractive molecules to be used as therapeutic agents, not only against tick-borne pathogens, but also to important human and animal disease-causing agents. On the other hand, interest in the therapeutic applications of antimicrobial peptides or their synthetic analogues is increasing due to the rise in resistance to commonly used antibiotics [4, 16].

Tick-borne flaviviruses (TBFVs) cause considerable disease and death worldwide, wherein infections are characterized by mild to severe neurological symptoms, such as meningitis and encephalitis [17, 18]. For Europe, Russia and up to the eastern coast of Japan, tick-borne encephalitis virus is considered as one of the most medically important arboviruses with 10,000 to 15,000 cases recorded each year [18, 19]. Since most TBFVs require at least a biosafety level 3 (BSL3) containment facility, the use of the naturally attenuated Langat virus (LGTV) provides a convenient BSL2 model of tick-borne encephalitis virus (TBEV) and other highly pathogenic TBFVs [17]. In this study, we investigated the virucidal activity of longicin P4 against LGTV, a member of TBEV serocomplex of the *Flaviviridae* family.

## Methods

### Cell culture and virus

Baby hamster kidney (BHK-21) cells (ATCC CCL-100) were maintained in Eagle’s minimum essential medium (EMEM) (Wako, Japan) containing 10 % fetal bovine serum (FBS) (Equitech, USA) and 1 % antibiotic/anti-mycotic (Nacalai Tesque, Japan), while HeLa cells (ATCC CCL-2) were maintained in Dulbecco’s modified Eagle’s medium (DMEM) (Nissui Pharmaceutical Co., Japan) supplemented with 10 % FBS, 1 % antibiotic/anti-mycotic and 1 % L-glutamine (Wako, Japan). Both cells were maintained at 37 °C under 5 % CO_2_ until use.

The LGTV TP21 used in this study was amplified in BHK cells and the virus stock titer was determined by focus forming assay as previously described [20] with some modifications. Briefly, serial 10-fold dilutions of the virus stock were plated on 1 × 10^5^ cells/well of BHK-21 cells in 24-well plates. The infected cells were overlaid with 1.5 % methylcellulose containing modified Eagle’s medium (MEM) (Gibco, USA) with 1 % FBS and 1 % antibiotic/anti-mycotic. Viral foci were detected by a primary antibody against Langat virus surface proteins (hyper immune mouse polyclonal IgG) followed by Alexa Fluor® 488 goat anti-mouse IgG (Invitrogen, USA), 3-4 days post-infection (dpi). The number of foci was counted using a fluorescence microscope and the titer of virus stock was expressed as Foci-Forming-Unit (FFU). The virus stock was then aliquoted and stored at -80 °C.

Human adenovirus 25 (ATCC VR-1103) was propagated in HeLa cells and the virus stock titer was quantitated by a 50 % tissue culture infectious dose (TCID_50_) assay as previously described [21] with some modifications. Briefly, serial 10-fold dilutions of the virus stock were plated (eight wells per dilution) on 1 × 10^4^ cells/well of HeLa cells in 96-well plates. Cytopathic effect was scored 6-7 dpi. The TCID_50_ was calculated as the inverse of the dilution at which 50 % of the wells showed cytopathic effect, calculated by the method of Reed & Muench [22].

### Ethical approval

The use of the animals in our experiments was in accordance with the approved guidelines from Animal Care and Use Committee of Kagoshima University (approval number VM 13007).

### Ticks and animals

Adult parthenogenetic (Okayama strain) *H. longicornis* ticks were used in this study. These ticks were maintained for several generations by feeding on the ears of Japanese white rabbits (Kyudo, Kumamoto, Japan) at the Laboratory of Infectious Diseases, Joint Faculty of Veterinary Medicine, Kagoshima University, Kagoshima, Japan. The rabbits were solely used for tick feeding and were not infected with any virus at any point during the conduct of this study.

### Peptides

Peptides were synthesized using a Perkin-Elmer Applied Biosystems 431 A Synthesizer with prederivatized polyethylene glycol polystyrene arginine resin (Sigma Genosys, Ishikari, Japan) and double coupling for residues. The reduced peptides were purified using reverse HPLC. The partial peptides are as follows: longicin P1 (QDDESDVPHVRVRRG 15 mer, Mw: 1764.8, pI: 5.43) and longicin P4 (SIGRRGGYCAGIIKQTCTCYR 21 mer, Mw: 2306.7, pI: 9.50). Peptide purity and integrity were assessed by MALDI-TOF Mass. The peptides were dissolved in normal saline (0.85 % w/v of NaCl) with a final concentration of 1 mmol/ml. The solutions were stored at -30 °C until use [15].

### Cell proliferation assay

The CellTiter 96® Non-Radioactive Cell Proliferation Assay System (Promega, USA) was used to examine the toxicity of longicin P1 and P4 peptides on BHK-21 cells as per manufacturer’s instructions. In brief, BHK-21 cells grown in EMEM were harvested and resuspended in a fresh medium at a final concentration of 1 × 10^5^ cells/ml*.* Fifty microliters of the cell suspension was dispensed into each well of a 96-well microtiter plate containing an equal volume of either the 2-fold serially diluted longicin P1 and P4 peptides or only the growth medium. After 72 h of incubation, dye solution was added to each well followed by 4 h of additional incubation. The reaction was then stopped by adding a solubilization solution, and absorbance was recorded at 570 nm in a microplate reader. Cytotoxicity was expressed as the percentage inhibition of cell growth (%) and was calculated as follows: Percentage cell growth inhibition (%) = (1–A/B) × 100, where A and B represent the absorbance value in the presence or absence of the peptide [14].

### Focus formation unit reduction assay

Antiviral activity of longicin peptides was determined by measuring the reduction in the number of viral foci. Briefly, BHK-21 cells were prepared in 24-well plates (1 × 10^5^ cells/ml). The infected cells were overlaid with 1.5 % methylcellulose containing MEM with 1 % FBS. Viral foci were detected by a primary antibody against Langat virus surface proteins (mouse polyclonal IgG) followed by a secondary antibody (Alexa Fluor® 488 goat anti-mouse IgG), three to four dpi. The number of foci was counted using a fluorescence microscope and the titer of virus was expressed as foci-forming-unit (FFU). The baseline for medium-treated infected cells was the mean of the viral foci number ± SD. Then the percentage of foci reduction (RF%) compared to medium-treated cells was calculated as follows: RF(%) = (C-T) × 100/C. Where, C is the mean of the number of foci in medium-treated wells (without peptide) and T is the mean of the number of foci in peptide-treated wells [23].

### Virucidal assay

Extracellular anti-LGTV activity of longicin P1 and P4 peptides was investigated by incubating 0.01 multiplicity of infection (MOI) of LGTV suspension with 1.25 μM longicin P1 or P4 for 2 h at 37 °C. For each assay, an EMEM-treated LGVT and an EMEM only control culture were included. Then, BHK-21 cells in 24-well plates were infected with the treated viral suspension for 1 h at 37 °C. Cells were washed with PBS to remove the unadsorbed viruses. Then, the plates were incubated at 37 °C for 3-4 days. Antiviral activity was determined by the reduction in number of viral foci [23]. For the dose-dependent virucidal assay, LGTV was treated with two-fold dilutions of longicin P4 starting from 0.16 to 2.5 μM for 2 h at 37 °C. On the other hand, to determine the time-dependent effect of longicin P4, LGTV was treated with 1.25 μM at different exposure times (0, 15, 30, 60, 120, 240 mins) at 37 °C. Foci forming unit reduction assay was used to determine the dose- and time-dependent antiviral effects of longicin P4 peptides.

The virucidal activity of longicin P4 was also tested against a non-enveloped virus by incubating 10 TCID_50_ of adenovirus suspension with 1.25 μM of longicin P1 or P4 for 2 h at 37 °C. For this assay, a DMEM-treated LGVT and a DMEM only control culture were included. Then, HeLa cells in 24-well plates were infected with the treated viral suspension for 1 h at 37 °C. Cells were washed with PBS to remove the unadsorbed viruses. The microplate was then incubated at 37 °C and observed daily under inverted microscope until CPE was more than 50 % in the virus group. Images were taken 6-7 days post-infection and at same time, respective supernatants were collected for titration.

### Prophylactic antiviral assay

The prophylactic antiviral assay was performed by treating BHK-21 cells in 24-well plates with EMEM or 1.25 μM of longicin P1 or P4 for 2 h at 37 °C prior to virus infection. After washing with PBS, cells were infected with 0.01 MOI of LGTV for 1 h for virus adsorption. Then cells were washed by PBS and overlaid with 1.5 % methylcellulose containing MEM with 1 % FBS. After 3-4 dpi, the antiviral activity was determined by foci reduction as described above [23].

### Post-adsorption antiviral assay

The antiviral activity of longicin peptides against intracellular replication of LGTV was performed by treating BHK-21 cells in 24-well plates with EMEM or 1.25 μM of longicin P1 or P4 after virus adsorption of 0.01 MOI of LGTV for 1 h at 37 °C. Unadsorbed viruses were washed with PBS and then the cells were treated with medium or 1.25 μM of peptides for 3-4 days. Antiviral activity was determined by viral foci reduction assay as described above [23].

### Virus yield reduction assay

Briefly, BHK-21 cells in 24-well plates were infected at a MOI of 0.01 with the LGTV pre-treated with longicin P1, P4 or medium for 2 h at 37 °C. After 1 h of viral adsorption at 37 °C, cells were washed with PBS to remove the unadsorbed viruses and consequently replaced with EMEM with 1 % FBS. Three days post-infection, supernatant from the infected cells were collected for titration [24].

For the adenovirus virucidal assay, virus yield was quantitated by a 50 % tissue culture infectious dose (TCID_50_) assay. Briefly, HeLa cells infected with adenovirus, which were previously treated with longicin P1, P4 and medium were sampled at 7 dpi. Supernatants were harvested and centrifuged to remove debris. Serial 10-fold dilutions of the supernatants were plated (five wells per dilution) on 1 × 10^4^ cells/well of HeLa cells in 96-well plates. Cytopathic effect was scored 6-7 days post-infection. The TCID_50_ was calculated as the inverse of the dilution at which 50 % of the wells showed cytopathic effect, calculated by the method of Reed and Muench [22].

### RNA interference (RNAi) and subsequent virus challenge

The PCR primers used for the synthesis of double-stranded RNA (dsRNA) are listed in Table 1. The *longicin* fragments were amplified by PCR from cDNA clones using oligonucleotides including T7-forward and T7-reverse primers to attach the T7 promoter recognition sites on both the forward and reverse ends. The firefly *luciferase* (*Luc*) was amplified from a vector DNA of pGEM-luc (Promega, Madison, WI, USA) through PCR using oligonucleotides containing T7-forward and T7-reverse primers. PCR products were purified using the GENECLEAN II kit (MP Biomedicals, Ohio, USA). The T7 RiboMAX Express RNA System (Promega) was used to synthesize dsRNA by in vitro transcription following the manufacturer’s protocol. Successful construction of dsRNA was confirmed by running 0.5 μl of the dsRNA products in a 1.5 % agarose gel in a TAE buffer. Microinjection of dsRNA was performed as previously described [25]. Briefly, 1 μg of *longicin* dsRNA in 0.5 μl of distilled water was injected into the hemocoel of unfed adult female ticks through the fourth coxae, while *Luc* dsRNA was injected in the control group. A total of 92 ticks per group were injected. After injection, the ticks were held for 18 h in a 25 °C incubator to check for mortality resulting from possible injury during injection. To initially confirm gene-specific silencing, 3 ticks from each group were collected at 0 and 4 days after injection (dai), and then total RNA was prepared for RT-PCR. RNA extraction was performed as previously described [25] and PCR was carried out using *longicin* and *actin* gene-specific primers (Table 2). PCR products were subjected to electrophoresis in 1.5 % agarose gel in a TAE buffer, and bands were visualized after staining the gel with ethidium bromide using Quantity One 1-D Analysis Software (Quantity One Version 4.5, Bio-Rad Laboratories, Milan, Italy). Positive confirmation of *longicin* silencing was monitored until 60 dai of *longicin* dsRNA.

**Table 1:** List of PCR primers used for the synthesis of double-stranded RNA

Primer name	Primer sequence
Longicin RNAi forward	CCTCATCTTCGTCCTTGTAG
Longicin RNAi reverse	ATTATGACGACACACATAAT
Longicin T7 forward	GGATCCTAATACGACTCACTATAGGCCTCATCTTCGTCCTTGTAG
Longicin T7 reverse	GGATCCTAATACGACTCACTATAGGATTATGACGACACACATAAT
Luc T7 forward	GTAATACGACTCACTATAGGGCTTCCATCTTCCAGGGATACG
Luc T7 reverse	GTAATACGACTCACTATAGGCGTCCACAAACACAACTCCTCC

**Table 2:** List of PCR primers used for the detection of *longicin* gene

Primer name	Primer sequence
Longicin forward	ATGAAGGTCCTGGCTGTTGC
Longicin reverse	CTACTTGCGGTAGCACGTGC
Hlβ-actin forward	ATCCTGCGTCTCGACTTGG
Hlβ-actin reverse	GCCGTGGTGGTGAAAGAGTAG

To further check the *longicin* gene silencing efficiency, expression analysis of the *longicin* mRNA was also performed through real-time PCR using THUNDERBIRD™ SYBR® qPCR Mix (TOYOBO) with a 7300 real-time PCR system (Applied Biosystems), as previously described [26]. Briefly, gene-specific primers were designed to target the *H. longicornis longicin* and ribosomal protein *L23* (internal control) genes*,* as shown in Table 3. Four-fold serial dilutions of cDNA of adult ticks were used to generate standard curves. The real-time PCR conditions were as follows: 95 °C for 10 min, 40 cycles of a denaturation step at 95 °C for 15 s, and an annealing/extension step at 60 °C for 60 s. The amount of *longicin* expressions was divided by the amount of *L23* expressions for both respective groups to obtain the normalized *longicin* expressions. Each sample was run in triplicates and the data were analyzed using the 7300 system SDS software (Applied Biosystems).

**Table 3:** List of real-time PCR primers used for the determination of *longicin* gene silencing efficiency

Primer name	Primer sequence
Longicin real-time forward	ACATGAAGGTCCTGGCTGTTG
Longicin real-time reverse	TCTCGTCATCTTGAGCTGCTG
L23 real-time forward	CACACTCGTGTTCATCGTCC
L23 real-time reverse	ATGAGTGTGTTCACGTTGGC

The percentage of gene silencing efficiency (%) of ticks injected with *longicin* dsRNA compared to *Luc* dsRNA-injected ticks was calculated as follows: Gene silencing efficiency (%) = (1–L_Long_/L_Luc_) × 100. Where, L_Long_ and L_Luc_ represent the normalized *longicin* expressions of *longicin* dsRNA-injected and *Luc* dsRNA-injected ticks, respectively [27].

Lastly, at 4 dai of *longicin* dsRNA, both *longicin gene*-silenced and *Luc gene*-injected ticks were challenged with LGTV (approximately 2.9 × 10^4^ffu/0.5 μl) via percoxal microinjection. Right after the challenge, the ticks were held for 18 h in a 25 °C incubator to check for mortality resulting from possible injury during injection. Thirty ticks per group were monitored for tick mortality for up to 35 dpi, while the remaining ticks per group were used for virus titration done at 0, 1, 3, 7, 14, 21, 28 dpi.

### Statistical analysis

Data were statistically analyzed using Student’s t–test wherein *P* values less than 0.05 were regarded as significant. All samples were tested at least in triplicate.

The Mantel-Cox log-rank test was also performed using GraphPad Prism software to determine significant difference in mortality (*P <*0.05) between control and *longicin* gene-silenced ticks challenged with LGTV.

## Results

### Cytotoxicity activity of the longicin P4 peptide

**Fig. 1:**
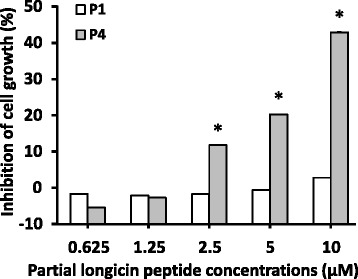
Cytotoxicity of longicin P1 and P4 against BHK-21 cells. MTT assay was used to evaluate the cytotoxicity of the peptides. Values are representative of triplicate samples and error bars indicate the range of values obtained. *p < 0.05, longicin P1 vs longicin P4

To eliminate the possibility that foci reduction was due to reduction in the number of viable host cells, we examined the cell growth inhibitory effect of partial peptides longicin P1 and P4 on BHK-21 cells (Fig. 1). Longicin P1 did not show any significant cytotoxicity on BHK-21 cells. In contrast, longicin P4 only demonstrated a non-significant cytotoxic effect at 1.25 μM concentration. Based on these results, the 1.25 μM concentration was used for both peptides in the succeeding tests.

### Antiviral effect of longicin P4 peptide against LGTV

**Fig. 2:**
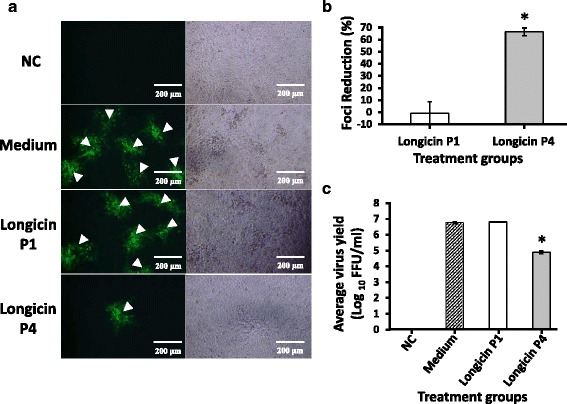
Virucidal effect of longicin P1 and P4 against Langat virus. **a** Fluorescence images of BHK-21 cells infected with Langat virus (TP-21) treated with medium only, 1.25 μM of P1 and P4 for 2 h at 37 °C. Arrowheads point to positive fluorescence FFU. **b** Foci reduction and **c** yield reduction assays were used to determine extracellular virucidal effect of longicin peptides. The percentage of foci reduction (%) was obtained by comparing against medium-treated cells maintained in parallel. All experiments were conducted in triplicates and error bars indicate the range of values. NC refers to cells with no treatment and no infection. *p < 0.05, longicin P4 vs longicin P1 or medium

Since defensin molecules accumulate in microbial membranes resulting in formation of pores in the targeted membrane, we checked first the extracellular virucidal activity of longicin peptides against LGTV. As shown in Fig. 2a, co-incubation of 1.25 μM longicin P4 with the virus at 37 °C for 2 h reduced the number of fluorescence-positive viral foci which is equivalent to a 70 % foci reduction (Fig. 2b). However, the same effect was not observed from longicin P1 treatment.

Likewise, to further support that longicin P4 has a virucidal activity against LGTV, we further checked if the longicin P4 can lower down the virus yield post-infection as compared to medium- and longicin P1-treated LGTV. As shown in Fig. 2c, cells infected with virus treated with longicin P4 produced almost two-fold lower titer as compared to cells infected with either medium- or longicin P1-treated virus. Although the difference may seem low, the virus titer of longicin P4 group corresponds to more than 90 % foci reduction in contrast to medium or longicin P1 treatment.

**Fig. 3:**
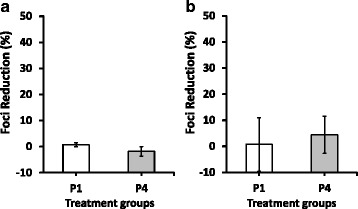
Prophylactic (**a**) and post-adsorption (**b**) antiviral effects of longicin P1 and P4 against Langat virus. Experiments were conducted in triplicates and error bars indicate the range of values. The percentage of foci reduction (%) was obtained by comparing against medium-treated cells maintained in parallel. *p < 0.05, longicin P4 vs longicin P1 or medium

In addition, to further support that the possible mechanism of action of longicin P4 against LGTV is through extracellular virucidal activity and exclude alternative possibilities, we also conducted prophylactic and post-adsorption antiviral assays. In the prophylactic antiviral assay, cells exposed to longicin P1 and P4 for two hours at 37 °C prior to virus infection showed statistically non-significant foci reduction at 0.76 and -1.820 %, respectively (Fig. 3a). Similarly, no significant antiviral activity was also recorded for both longicin peptides against LGTV in the post-adsorption antiviral assay, wherein, longicin P1 and P4 showed 0.71 and 4.4 % foci reduction, respectively (Fig. 3b).

**Fig. 4:**
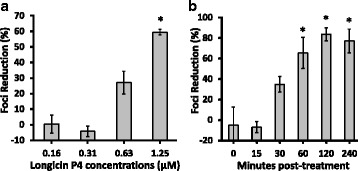
Dose-dependent (**a**) and time-dependent (**b**) virucidal effects of longicin P4 against Langat virus. Experiments were conducted in triplicates and error bars indicate the range of values. The percentage of foci reduction (%) was obtained by comparing against medium-treated cells maintained in parallel. *p < 0.05, as compared to the lowest concentration or to 0 min

Moreover, after establishing that co-incubation of longicin P4 with LGTV can successfully reduce foci formation and virus yield, we then checked the dose-dependent and time-dependent virucidal capacity of longicin P4. As shown in Fig. 4a, 1.25 μM of longicin P4 can significantly produce more than 50 % foci reduction against LGTV, while the lowest concentration to show a significant foci reduction was 0.65 μM. Likewise in Fig. 4b, we can also observe that before adding to the cells, at least 30 min of close contact between the virus and longicin P4 at 37 °C is required to achieve significant foci reduction and the optimum virucidal activity can be achieved at 2 h treatment.

### Antiviral effect of longicin P4 peptide against human adenovirus

**Fig. 5:**
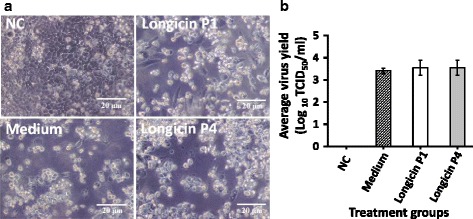
Virucidal activity of longicin P1 and P4 against adenovirus. **a** Images of HeLa cells infected with human adenovirus 25 treated with medium only, 1.25 μM of longicin P1 and P4 for 2 h at 37 °C. Images were taken 7 dpi (200xmagnification). **b** TCID_50_ was used to determine the virus yield titers of the collected supernatants of the treatment groups. Data expressed as means ± SD

Our results showed that longicin P4 has a virucidal activity against LGTV and it is supported by previous findings that suggest that cationic antimicrobial peptides such as longicin P4 can only target pathogens possessing a membrane [10, 11]. Thus, to check if longicin P4 can only exert antiviral activity against membrane-bound or enveloped viruses, we determined its virucidal activity against a non-enveloped virus. As shown in Fig. 5a, co-incubation of 1.25 μM of longicin P4 and P1 failed to reduce adenovirus infectivity leading to a successful viral infection and marked cell death. In addition, no significant difference can be observed in the virus yield from medium-, P1- and P4-treated adenovirus (Fig. 5b).

### Effect of *longicin* gene silencing in LGTV replication in adult *H. longicornis*

**Fig. 6:**
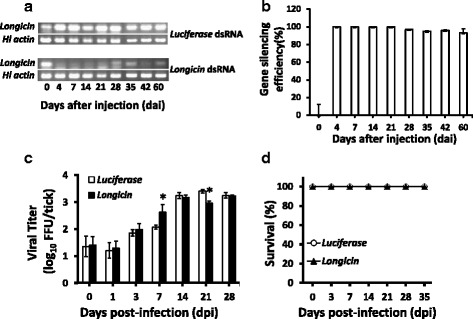
Effect of *longicin* silencing in tick mortality and virus titer. **a** To confirm gene-specific silencing, 3 ticks from each group were collected at 0, 4, 7, 14, 21, 28, 35, 42 and 60 dai of dsRNA. Initial confirmation of *longicin* silencing was carried out through RT-PCR and gel electrophoresis (**a**), while gene silencing efficiency was determined by real-time PCR (**b**). Virus titers (**c**) and tick survival (**d**) were monitored after injecting LGTV on 4-day *luciferase* dsRNA- or *longicin* dsRNA-inoculated ticks. Values for mortality (*n* = 30 ticks per group) were expressed as the percentage of live ticks remaining to the number of ticks used at the beginning of the experiment in different time courses. Significant difference (*p < 0.05) was determined using the Mantel-Cox log-rank test, while error bars in virus titers indicate SD in mean values of 5 ticks. *p < 0.05, *luciferase* vs *longicin*

To evaluate the importance of longicin in the innate immunity of *H. longicornis* adult ticks against LGTV, gene silencing through RNAi was performed. Ticks were individually injected with either *longicin* dsRNA or with *Luc* dsRNA for the control group. Silencing of *longicin* gene was confirmed visually by regular RT-PCR and gel electrophoresis (Fig. 6a). In addition, gene silencing efficiency was also determined using real-time PCR (Fig. 6b), wherein more than 90 % of *longicin* mRNA reduction can be observed for at least 60 days after microinjection of *longicin* dsRNA.

Although mean virus titers from whole ticks (Fig. 6c) demonstrate significant differences at 7 and 21 dpi between *longicin* dsRNA and *Luc* dsRNA injected ticks, the titers for both groups eventually equalized at the end of the observation period. In addition, no significant difference was observed in tick mortality on both groups after 35 dpi (Fig. 6d).

## Discussion

Several studies have already shown the antimicrobial, fungicidal, and parasiticidal activities of longicin and one of its synthetic partial analogs (longicin P4). A previous study has shown that some known defensins have a common motif (G^3^RRGG^5^) which could be related to their antiparasitic activity [28]. Such motif was also found in longicin P4 suggesting that this motif is the one responsible for its antiparasitic activity [15, 29]. Since longicin P4 has been shown to possess antimicrobial, fungicidal, and parasiticidal properties, we decided to test the peptide for its antiviral activity against a tick-borne flavivirus.

In this study, our data show that co-incubation treatment of LGTV with longicin P4 at 1.25 μM concentration prior to infection resulted in significant foci reduction. And to clarify if this antiviral activity is also present in the full-length (FL) longicin peptide, we also checked the antiviral capacity of the FL longicin against LGTV. Initially, we determined first the non-cytotoxic concentration of FL longicin on BHK cells (see Additional file 1a) and used this concentration as the treatment concentration. As expected, FL longicin also exhibited an almost 40 % viral foci reduction (see Additional file 1b).

On the other hand, the virus yield from cells infected with virus co-incubated with longicin P4 produced lower titer as compared to cells infected with either medium or longicin P1 treated virus, resulting in more than 90 % foci reduction as compared to medium or longicin P1 treatment.

The lower virus yield from cells infected with virus co-incubated with longicin P4 further confirm that the virucidal activity of longicin P4 is extracellular, through close contact, as previously shown in another study that longicin P4 impairs parasite membranes (*Toxoplasma gondii*) resulting in the reduction of infection in cells [15]. In addition, the failure of longicin P4 to significantly reduce foci formation in the prophylactic and post-adsorption antiviral assays clearly supports that the antiviral activity of the peptide is exerted through extracellular inactivation of the virus particle.

LGTV, being an enveloped virus, has an outer coating that is composed of a lipid bilayer. Viral envelope can be a target for longicin as defensin molecules accumulate in microbial membranes resulting in the formation of pores in the targeted membrane [30]. However, additional experiments to show the binding between extracellular virus and longicin P4 are needed to fully establish the exact mechanism of membrane targeting of longicin in enveloped viruses. Nevertheless, to confirm that the effect of longicin P4 may only be limited to membrane-bound targets, a common trait for cationic antimicrobial peptides, we tested its virucidal activity against human adenovirus 25, a non-enveloped virus. As shown in Fig. 5, longicin P4 failed to inhibit virus replication, thus supporting the claims that although cationic antimicrobial peptides have diverse targets, their activity is generally limited to targets with membranes [11, 29].

However, previous findings by Smith and Nemerow [31], showed that human α-defensin can inhibit adenovirus infection by directly binding to non-enveloped adenoviral capsid, inhibiting virus disassembly. Such binding ultimately leads to inhibition of endosomal membrane penetration during cell entry. This finding further explains that the virucidal activity of longicin P4 may only be limited to enveloped viruses and the synthetic peptide does not target the capsid of adenovirus. Likewise, it is also possible to suppose that longicin P4 can directly bind on the membrane of LGTV without disrupting the viral envelope, and in effect inhibits the binding of the virus to cellular receptors for viral entry. However, this mechanism remains to be elucidated.

Lastly, to fully elucidate the importance of longicin in the innate immunity of *H. longicornis* ticks against LGTV, gene silencing through RNAi was performed. After successfully silencing the *longicin* gene, ticks were challenged with LGTV via microinjection. Preliminary results on the effect of *longicin* gene silencing on virus titer show that significant difference can be observed at 7 and 21 dpi. At 7 dpi, *longicin* gene-silenced ticks produced higher viral titer as compared to *luc* dsRNA injected ticks, which may be attributed to *longicin* gene silencing. However, at 21 dpi, *longicin* gene-silenced ticks showed lower viral titer as compared to the control. Although a relatively high gene silencing efficiency (more than 90 %) can be observed for at least 60 dai, the lower viral titer in the *longicin* gene-silenced group at 21 dpi remains to be answered, thus suggesting that the effect of *longicin* gene silencing on the survival dynamics of LGTV in vivo still remains unclear. In addition, a complete knockdown of the *longicin* gene may also be needed to fully assess the function of longicin against LGTV in vivo.

On the other hand, for the whole duration of the study, no significant difference in mortality was observed on both *longicin* gene-silenced and *Luc dsRNA-injected* ticks. Failure to observe any significant effect on tick mortality on the *longicin*-silenced ticks, may suggest that the activity of longicin in the tick is not related to the control LGTV infection or the virus model used for this study may not be suitable to test our hypothesis. Likewise, the sturdiness of ticks against any harmful effect due to LGVT infection may be expected since ticks act as highly efficient reservoirs of flaviviruses [32]. Moreover, the failure of *longicin* silencing to affect the tick’s resistance to LGTV suggests that the peptide’s activity may only be effective in vitro*.* As previously observed, activities of antimicrobial peptides can be undesirably affected by various relevant factors present in vivo and these may include proteases, polyanions and high mono- and divalent cation concentrations [12].

## Conclusion

In summary, we have successfully established the extracellular virucidal activity of longicin P4 against LGTV in vitro, and to our knowledge, this is the first report of an antiviral activity of a native or synthetic antimicrobial peptide derived from *H. longicornis*. However, the role of the endogenous tick longicin in the antiviral defense of *H. longicornis* still remains to be demonstrated.

## Additional file

Additional file 1:
**Virucidal effect of full-length (FL) longicin against LGTV.** Foci reduction assay was used to determine the extracellular virucidal effect of baculovirus-expressed FL longicin peptide using 0.5 nm concentration. Based from the cell proliferation assay (a), 0.5 nM of FL longicin showed no significant cytotoxicity on BHK cells that may affect the result of the foci reduction assay. (b) The percentage of foci reduction (%) was obtained by comparing against medium-treated cells maintained in parallel. All experiments were conducted in triplicates and error bars indicate the range of values. (PDF 109 kb)

## Abbreviations

dsRNA: double-stranded RNA
*Luc*: firefly *luciferase*
PBS: phosphate buffered saline
RNAi: RNA interference
