# Supplementary figures and images for: Virucidal activity of Haemaphysalis longicornis longicin P4 peptide against tick-borne encephalitis virus surrogate Langat virus
Source: Parasit Vectors. 2016 Feb 2;9:59. doi: 10.1186/s13071-016-1344-5 (PMC4736483; doi:10.1186/s13071-016-1344-5)

**A**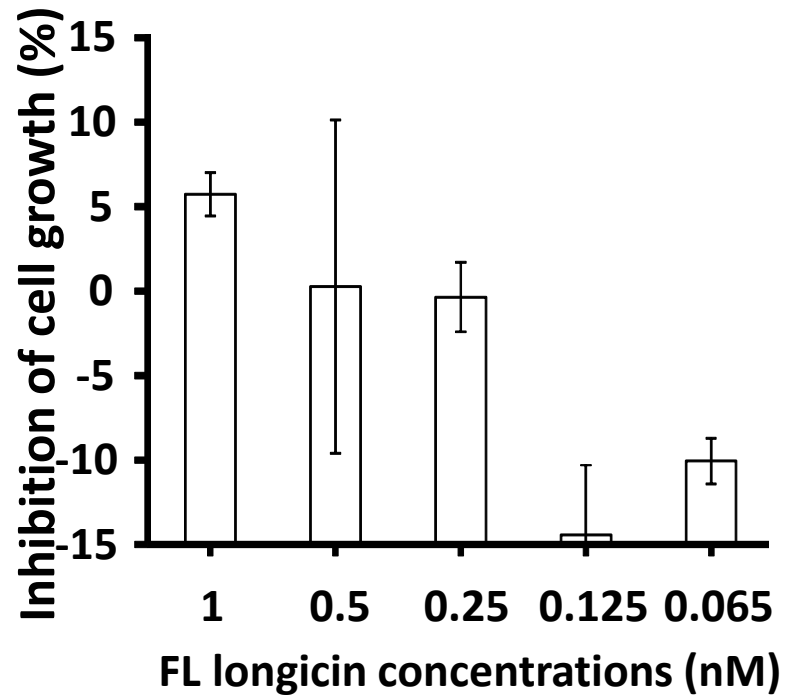**B**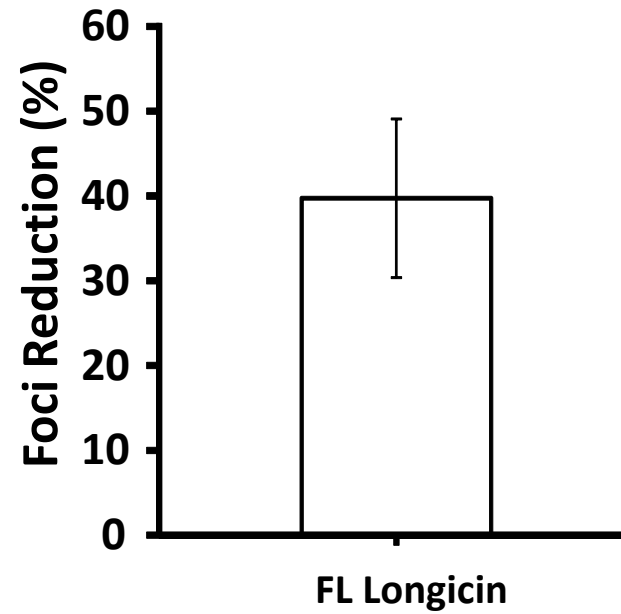

Supplement: Additional file 1: — Virucidal effect of full-length (FL) longicin against LGTV. Foci reduction assay was used to determine the extracellular virucidal effect of baculovirus-expressed FL longicin peptide using 0.5 nm concentration. Based from the cell proliferation assay (a), 0.5 nM of FL longicin showed no significant cytotoxicity on BHK cells that may affect the result of the foci reduction assay. (b) The percentage of foci reduction (%) was obtained by comparing against medium-treated cells maintained in parallel. All experiments were conducted in triplicates and error bars indicate the range of values. (PDF 109 kb) [file 13071_2016_1344_MOESM1_ESM.pdf]
